# Traumatic Cardiac Injury: Ventricular Perforation Caught on CT

**DOI:** 10.1155/2016/9696107

**Published:** 2016-06-12

**Authors:** Behrad Golshani, Paul Dong, Scott Evans

**Affiliations:** Department of Radiology, University of California, Davis Medical Center, Sacramento, CA 95817, USA

## Abstract

Myocardial rupture is a rare imaging diagnosis given its clinical severity and high mortality. Early findings should be promptly communicated to the trauma service to ensure immediate intervention. We present a rare case of blowout perforation of the right ventricle which was prospectively diagnosed on computed tomography (CT) leading to emergent operative repair. The patient subsequently survived and was discharged after a lengthy hospital course.

## 1. Introduction

Cardiac trauma encompasses a wide spectrum of injury ranging from contusion to frank perforation. Contrast enhanced chest CT is a powerful diagnostic tool in patients with suspected cardiac trauma and the appropriate findings can help establish or further pursue the appropriate diagnosis. Cardiac rupture is only rarely depicted on CT due to its immediate grave prognosis; however, it is an entity which should not be neglected as timely management is critical.

## 2. Case Report

**Figure 1:**
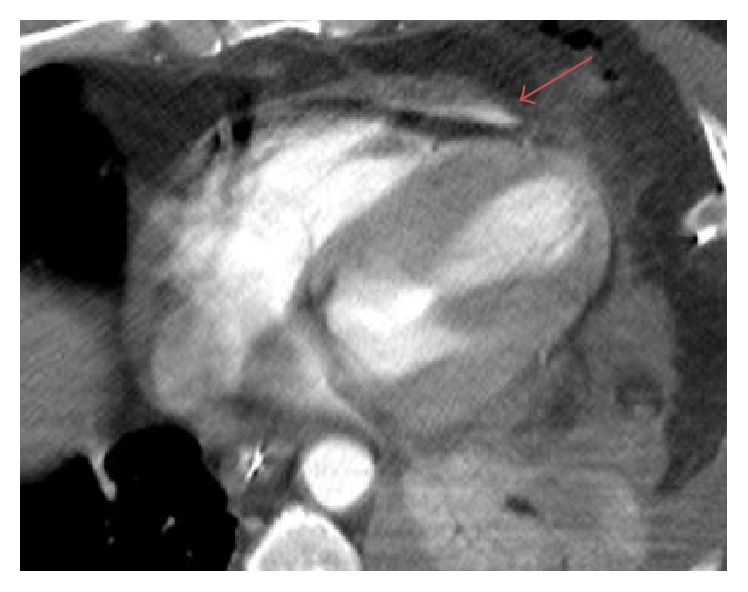
64-year-old male diagnosed with traumatic right ventricular rupture. Routine axial contrast enhanced CT demonstrates active extravasation of intravenous contrast into the anterior pericardium (arrow).

**Figure 2:**
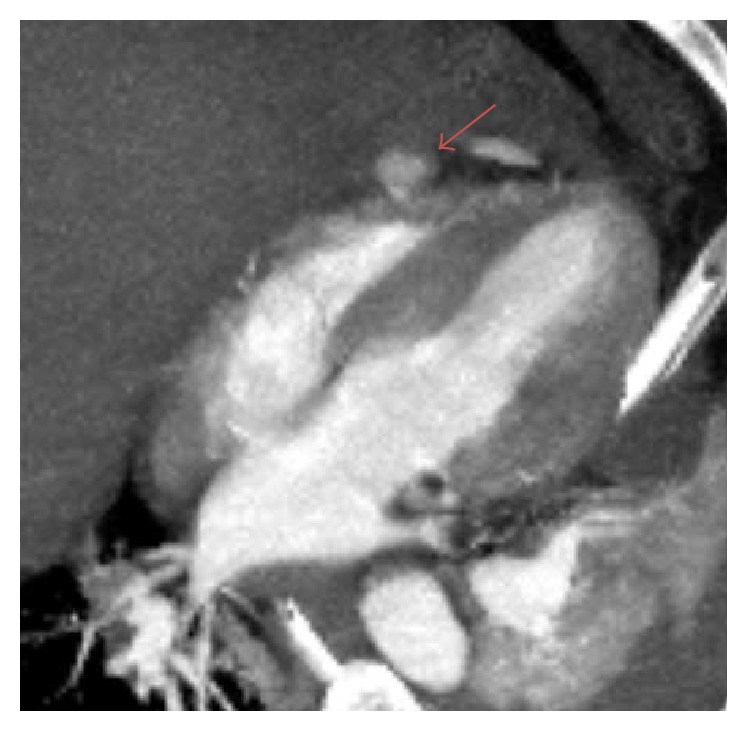
64-year-old male diagnosed with traumatic right ventricular rupture. Reformatted, axial oblique contrast enhanced CT through the right and left ventricles demonstrates outpouching of contrast extending from the right ventricle (arrow).

**Figure 3:**
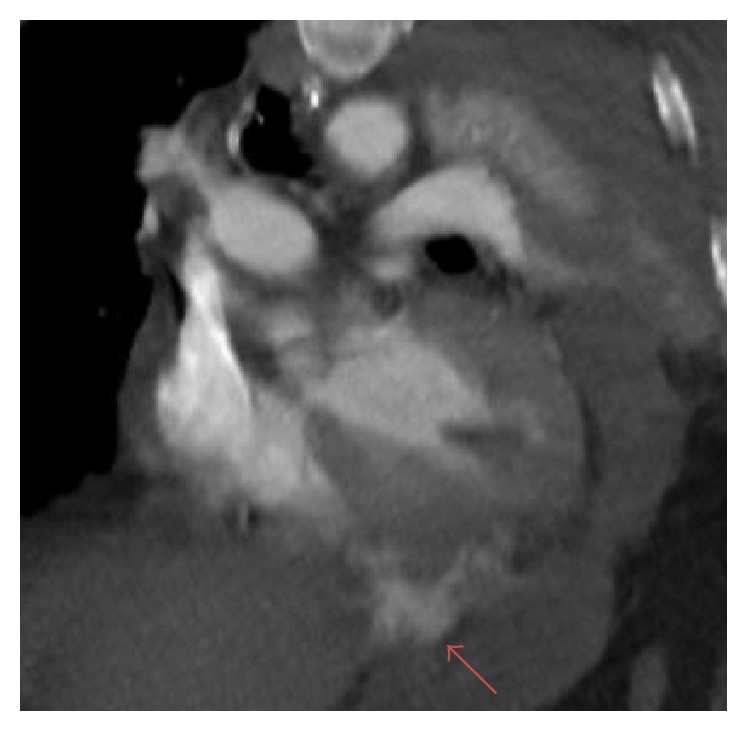
64-year-old male diagnosed with traumatic right ventricular rupture. Reformatted, coronal oblique contrast enhanced CT through the right and left ventricles demonstrates contrast extending from the right ventricle into the pericardium and intraperitoneal space (arrow).

**Figure 4:**
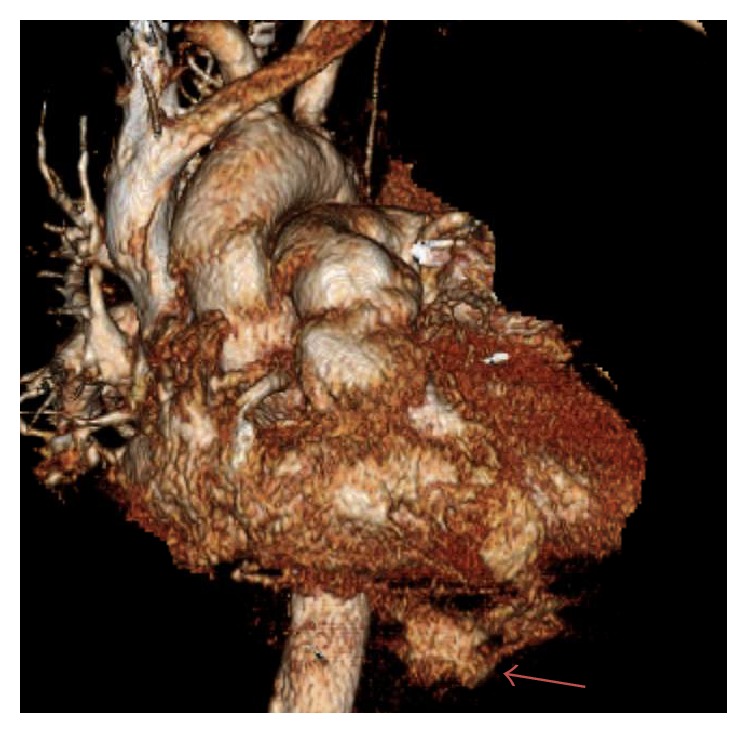
64-year-old male diagnosed with traumatic right ventricular rupture. 3D reformatted image from a routine contrast enhanced chest CT demonstrates contrast extravasation emanating from the right ventricle (arrow).

**Figure 5:**
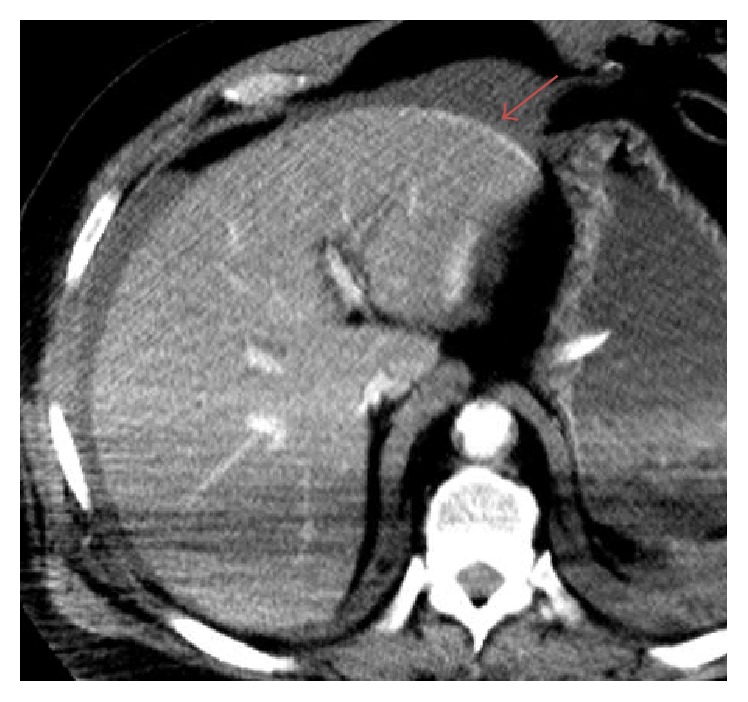
64-year-old male diagnosed with traumatic right ventricular rupture. Routine axial contrast enhanced CT demonstrates extravasated contrast communicating with the intraperitoneal space and lining the liver (arrow).

A 64-year-old male with no significant past medical history was brought by ambulance to the emergency department with severe chest pain and shortness of breath after a 1400-pound container fell on his chest from a height of three feet. Patient was noted to be hypotensive in route and had a recorded blood pressure of 88/45 with a heart rate of 135. Routine axial contrast enhanced CT of the chest demonstrated contrast extravasation into the pericardium (Figure 1). Multiplanar and 3D reformation demonstrated contrast tracking from the right ventricle (Figures 2, 3, and 4). Contrast was also seen tracking along the peritoneal lining surrounding the liver (Figure 5). Findings were prospectively interpreted as concerning for cardiac injury. The patient subsequently underwent cardiac arrest and was taken emergently to the operating room. Per operative report, there was traumatic compression injury with blowout perforation of the right ventricular apex. The right ventricular wall rupture was repaired and the patient received 28 units of packed red blood cells and 14 units of fresh frozen plasma intraoperatively. Multiple other traumatic, noncardiac findings were also present on CT, including, but not limited to, diaphragmatic rupture, liver laceration, and multiple rib and spine fractures. The patient was subsequently discharged after a prolonged hospital stay to a subacute nursing facility.

## 3. Discussion

Greater than 900,000 cases of cardiac trauma are reported in the United States each year [1]. Motor vehicle accidents are the most common mechanism, with crush injury reported as the second most common cause. Injury is classified as penetrating and blunt or nonpenetrating with the latter being more common [1].

Blunt cardiac injury ranges from contusion to myocardial perforation. Injury commonly occurs at the right ventricular free wall given its anterior location within the thorax. Reported incidence of myocardial rupture in patients presenting to the emergency department ranges from 0.3 to 1.1% [2]; however, it has been reported to be as high as 36–65% on autopsy of chest trauma patients, indicating that a vast majority of myocardial rupture results in near immediate death [2]. A series of 32 patients with cardiac rupture demonstrated an average age of 39.5 years and a near 2 : 1 male-to-female ratio [3].

Patients who have undergone blunt cardiac trauma present somewhat of a diagnostic dilemma. Clinical presentation may range from nonspecific to mimicking myocardial infarction to sudden death depending on severity of injury. Isolated EKG and cardiac enzyme markers are often not helpful in distinguishing myocardial ischemia from that of mild traumatic injury, although studies have found that troponin T and troponin I, in particular, may be useful for risk stratification [4, 5]. Emergent echocardiography plays an important role in the traumatic setting and may demonstrate findings suggestive of cardiac injury, such as abnormal myocardial echogenicity and wall motion abnormalities. An adequate window, however, may be challenging to obtain given the presence of supportive devices. CT is, therefore, considered a vital diagnostic tool in evaluating patients with blunt and penetrating thoracic trauma given the high sensitivity for cardiac and associated injuries such as pneumothorax and regional fractures [1].

The most specific finding on imaging suggestive of blunt cardiac trauma is pericardial effusion of varying density which is the CT equivalent of hemopericardium. CT has a reported sensitivity of 100% and a specificity of greater than 96% [1] in the diagnosis of hemopericardium. Visualized disruption of the pericardium, ventricular herniation, and/or hemothorax may be seen with pericardial injury/rupture. Myocardial rupture can be demonstrated by discontinuity of the myocardial wall, visualized communication of the ventricle or atrium with the pericardium, acute pericardial effusion, and active extravasation of contrast into the pericardium.

Management for cardiac trauma varies greatly with the severity of injury. Contusion may be managed conservatively, while myocardial rupture would necessitate emergent surgery for wall repair. Once identified, surgical repair is relatively straightforward. Mortality, however, is exceedingly high. A retrospective 5-year review of cardiac trauma cases reported an 81% mortality rate in patients with cardiac rupture [3].

The differential diagnosis primarily includes pericardial hematoma, pericardial effusion, and pericarditis. Radiograph is nonspecific for distinguishing these entities from traumatic cardiac rupture and may demonstrate enlargement of the cardiac silhouette. Pericardial hematoma may demonstrate high density pericardial fluid; however, it would not be expected to demonstrate active extravasation of contrast. Pericardial effusion may demonstrate an increased amount of simple, nonenhancing fluid within the pericardium. Chronic pericarditis may demonstrate calcification which should be differentiated from extravasation of intravenous contrast based on density and distribution.

## 4. Conclusion

CT is a vital diagnostic tool for patients with blunt and penetrating cardiac trauma. The vast majority of patients with cardiac rupture are not imaged given the immediate grave prognosis at the time of injury. Active extravasation of contrast, myocardial disruption, and visualized communication between the ventricle and pericardium may signal this rare imaging diagnosis.
